# Glutathione-Related Responses to Hyperglycemic and Hyperosmotic Stress in Cultured Human Retinal Pericytes and Endothelial Cells: An Exploratory Study

**DOI:** 10.3390/biomedicines14092104

**Published:** 2026-09-18

**Authors:** Ecaterina Pavlovschi, Felicia Darii, Valeriana Pantea, Ștefan Curtev, Cristina Trocin, Serghei Curlat, Dan-Cristian Ușurelu, Tatiana Mărițoi, Olga Tagadiuc

**Affiliations:** 1 Department of Biochemistry and Laboratory Medicine, Nicolae Testemițanu State University of Medicine and Pharmacy, Chisinau MD-2004, Moldova; ecaterina.pavlovschi@usmf.md (E.P.); cristinatn725@gmail.com (C.T.); sergiu.curlat@usmf.md (S.C.); dan.usurelu040599@gmail.com (D.-C.U.); olga.tagadiuc@usmf.md (O.T.); 2 Biochemistry Laboratory, Nicolae Testemițanu State University of Medicine and Pharmacy, Chisinau MD-2004, Moldova; valeriana.pantea@usmf.md (V.P.); kurtev.stefan.97@gmail.com (Ș.C.); 3 Morphology Laboratory, Nicolae Testemițanu State University of Medicine and Pharmacy, Chisinau MD-2004, Moldova; tatiana.maritoi@usmf.md

**Keywords:** diabetic retinopathy, human retinal pericytes, retinal endothelial cells, hyperglycemia, hyperosmotic stress, glutathione, glutathione peroxidase, γ-glutamyl transferase

## Abstract

**Background/Objectives:** Hyperglycemic and hyperosmotic stress may disturb glutathione-dependent antioxidant defenses in retinal vascular cells. This exploratory in vitro study evaluated glutathione-related biochemical endpoints in separate human retinal pericyte and endothelial-cell experiments and to identify candidate endpoints for independent validation. **Methods:** Passage-3 human retinal pericytes and endothelial cells were exposed for 24 h to five conditions: 5.5 mmol/L glucose (control), 25 or 50 mmol/L glucose, and 5.5 mmol/L glucose supplemented with 19.5 or 44.5 mmol/L mannitol. Glutathione peroxidase (GPx), glutathione reductase (GR), total glutathione, reduced glutathione (GSH), oxidized glutathione (GSSG), the GSH/GSSG ratio, glutathione S-transferase (GST), and gamma-glutamyl transferase (GGT) were measured in a mixture of culture medium and cell lysate. Each condition comprised 10 technical replicate wells from one culture experiment; the cell types were examined in separate, unmatched runs. **Results:** In the pericyte experiment, GPx and GR decreased across several glucose and mannitol conditions, and GGT increased by 39–72%. In the separate endothelial experiment, GGT was the dominant response (eta squared = 0.763), increasing by 66–116%; GGT at 50 mmol/L glucose was 30% higher than the corresponding 44.5 mmol/L mannitol condition. **Conclusions:** The study identifies GGT as the principal candidate endpoint for biological replication and GPx and GR as additional candidates in pericytes. It does not support comparisons between cell types. All findings are preliminary because they derive from technical wells in single, unmatched experiments.

## 1. Introduction

Diabetic retinopathy (DR) is a neurovascular complication of diabetes whereby the metabolic disturbances cause progressive damage to the retinal microvasculature. Hyperglycemia triggers an interconnected cascade of pathogenic mechanisms, including advanced glycation, protein kinase C activation, polyol pathway flux, mitochondrial dysfunction, inflammatory signaling, and overproduction of reactive oxygen species (ROS). These result in redox imbalances that affect the whole retinal neurovascular unit rather than a single type of vascular cell population [1,2,3,4,5].

Pericytes and endothelial cells are anatomically and functionally interconnected within the retinal capillary network. Pericytes maintain the vessel structure, control endothelial viability and function and also contribute to angiogenic signaling. The loss of pericytes is an early histopathological finding in DR. Endothelial cells constitute the inner blood–retinal barrier and are therefore exposed directly to metabolic disturbances. Because the two cell types differ in metabolic programs and stress susceptibility, a biomarker response detected in one compartment cannot automatically be generalized to the other [6,7,8,9,10].

The glutathione system is central to retinal antioxidant defense. GPx uses GSH to reduce peroxides, producing GSSG. GR reconstitutes GSH from GSSG using NADPH. GST conjugates electrophilic substrates to GSH, and GGT supports extracellular glutathione turnover and amino-acid salvage. GSH and GSSG concentrations, together with the GSH/GSSG ratio, provide complementary information on cellular redox status, although these derived measures are mathematically interdependent [11,12,13,14]. Alterations in glutathione homeostasis have been implicated in diabetic retinal injury, but glutathione-centered screening across high-glucose and mannitol conditions remains incompletely characterized in these human retinal vascular cell models [15,16,17,18].

High-glucose culture increases both substrate availability and medium osmolarity. Mannitol is commonly added to normoglycemic medium as an osmotic comparator, but it should not be interpreted as a biologically inert substitute: hyperosmolarity triggers adaptive and injury pathways, and mannitol itself may influence cell signaling [19,20]. Consequently, a glucose-specific effect is most convincingly supported by a difference between a high-glucose group and its osmotically matched mannitol condition, not solely by a difference from normoglycemic control.

The present study aimed to characterize treatment-associated glutathione responses within separate human retinal pericyte and endothelial-cell experiments exposed to high-glucose or mannitol-associated hyperosmotic conditions. The objective was to identify endpoints and glucose–mannitol contrasts that merit independent biological replication. No direct comparison between cell types was planned because the experiments were conducted in separate, unmatched runs.

## 2. Materials and Methods

### 2.1. Study Setting, Funding, and Ethics

The experiments were conducted in 2025–2026 in the Biochemistry Laboratory and at the Chair of Biochemistry and Clinical Biochemistry, Nicolae Testemițanu State University of Medicine and Pharmacy, Republic of Moldova. This work was conducted as part of the project “Study of the Impact of Hyperglycemic Shock on Pericytes in Metabolic Syndrome” (code 25.80012.8007.01TC), funded by the National Agency for Research and Development of the Republic of Moldova. The institutional Research Ethics Committee approved the protocol (approval no. 3, reference no. 86, 23 September 2025).

### 2.2. Cell Culture

Human retinal pericytes (HRPCs; catalogue P10869, Innoprot, Spain) were expanded in poly-L-lysine-coated culture flasks at 37 °C in a humidified atmosphere containing 5% CO_2_. Cells were maintained in Innoprot Pericyte Medium (P60121) containing 2% fetal bovine serum, 1% pericyte growth supplement, and 1% penicillin/streptomycin.

Human retinal endothelial cells (HRECs; catalogue P10880, Innoprot, Spain) were expanded under the supplier-recommended conditions in endothelial basal medium (P60104) containing 5% fetal bovine serum, 1% endothelial cell growth supplement, and 1% penicillin/streptomycin solution (all from Innoprot, Spain). Passage-3 cells were used for seeding and for all experimental exposures in both cell-culture experiments.

At approximately 80% confluence, cells were washed with phosphate-buffered saline, detached with 0.25% trypsin-EDTA, centrifuged at 500 rpm for 5 min, counted, and resuspended in the appropriate culture medium. Cells were seeded into flat-bottom 96-well plates at 1 × 10^5^ cells per well in 100 µL and allowed to adhere for 24 h at 37 °C.

### 2.3. Hyperglycemic and Mannitol-Associated Hyperosmotic Exposures

After the 24 h adherence period, cells remained in the 96-well plates and the medium was replaced with the assigned exposure medium: (I) 5.5 mmol/L glucose (control); (II) 25 mmol/L glucose; (III) 50 mmol/L glucose; (IV) 5.5 mmol/L glucose plus 19.5 mmol/L mannitol; or (V) 5.5 mmol/L glucose plus 44.5 mmol/L mannitol. Glucose was diluted from a 400 mg/mL stock and mannitol from a 150 mg/mL stock. All exposures lasted 24 h. Cultures were inspected with an inverted-light microscope (Nikon Corporation, Tokyo, Japan), equipped with NIS-Elements software—MShot Image Analysis System, Version V1.1.3, immediately before treatment and at the end of exposure.) immediately before treatment and at the end of exposure. No fluorescent live-cell labeling was performed, and microscopy was not analyzed as a quantitative endpoint.

The two mannitol concentrations were selected to match the nominal additional solute concentration in the 25 and 50 mmol/L glucose groups, respectively. Thus, the 25 mmol/L glucose group was compared with 5.5 mmol/L glucose plus 19.5 mmol/L mannitol, and the 50 mmol/L glucose group with 5.5 mmol/L glucose plus 44.5 mmol/L mannitol. These conditions are described as mannitol-associated hyperosmotic comparators rather than pure osmotic controls because measured osmolality was not reported and direct cellular effects of mannitol cannot be excluded [19,20].

### 2.4. Sample Collection

At the end of exposure, the conditioned medium from each well was transferred to an individual Eppendorf tube and combined with the corresponding cell lysate from that well. The resulting medium-cell-lysate mixture was subjected to repeated freeze–thaw cycles before biochemical analysis. Each condition comprised 10 technical replicate wells from one culture experiment. The wells were not independent biological replicates. Coded samples were stored at −40 °C until biochemical analysis.

### 2.5. Biochemical Endpoints

Eight endpoints were evaluated in the medium-cell-lysate mixture using standardized spectrophotometric procedures adapted to 96-well microplates: GPx, GR, reduced GSH, total glutathione, GSSG, the GSH/GSSG ratio, GST, and GGT. GPx activity was determined from the consumption of reduced glutathione during peroxide reduction, whereas GR activity was assessed from the NADPH-dependent reduction in oxidized glutathione. Reduced and total glutathione were quantified using thiol-dependent colorimetric reactions; GSSG and the GSH/GSSG ratio were obtained from the corresponding reduced and oxidized glutathione measurements. GST activity was determined from the rate of glutathione conjugation with an electrophilic substrate. GGT activity was assessed from cleavage of a chromogenic gamma-glutamyl substrate.

The procedures followed Gudumac et al. [21], with microplate adaptations of the methods reported by Andronache et al. for GPx, GR, and GSH [22,23,24] and by Tagadiuc et al. for GST [25]. GGT activity was measured with the Gamma-GT Plus SL kit (ELITech Clinical Systems SAS, Sees, France, LOT 25-0048) according to the manufacturer’s instructions. Absorbance was recorded with a Synergy H1 Hybrid Reader or PowerWave HT spectrophotometer (BioTek Instruments, Winooski, VT, USA, on a Cytation 5 with Gen5 software, version 3.15.15) in the Biochemistry Laboratory of Nicolae Testemitanu State University of Medicine and Pharmacy, and assay values were calculated according to the cited protocols.

### 2.6. Statistical Analysis

Analyses were performed in IBM SPSS Statistics version 23.0 (IBM Corp., Armonk, NY, USA). Data are summarized as mean ± standard deviation. Distributional shape was examined within each condition using the Shapiro–Wilk test, and homogeneity of variance was assessed with Levene’s test. Welch’s one-way analysis of variance and Games–Howell post hoc comparisons were used for exploratory analysis within each cell type because heterogeneity of variances was common. Kruskal–Wallis tests served as sensitivity analyses.

Analyses were conducted separately within the pericyte and endothelial-cell experiments. No inferential comparison between cell types and no cell-type-by-condition interaction test were performed because the experiments were conducted in separate, unmatched runs.

## 3. Results

All numerical comparisons below concern 10 technical replicate wells per condition from one culture experiment for each cell type. The wells were not independent biological replicates, and results from the two experiments were not compared statistically. Detailed means, dispersion, omnibus tests, effect sizes, and exploratory pairwise comparisons are reported in Table 1, Table 2 and Table 3.

### 3.1. Pericyte Response Profile

**Figure 1:**
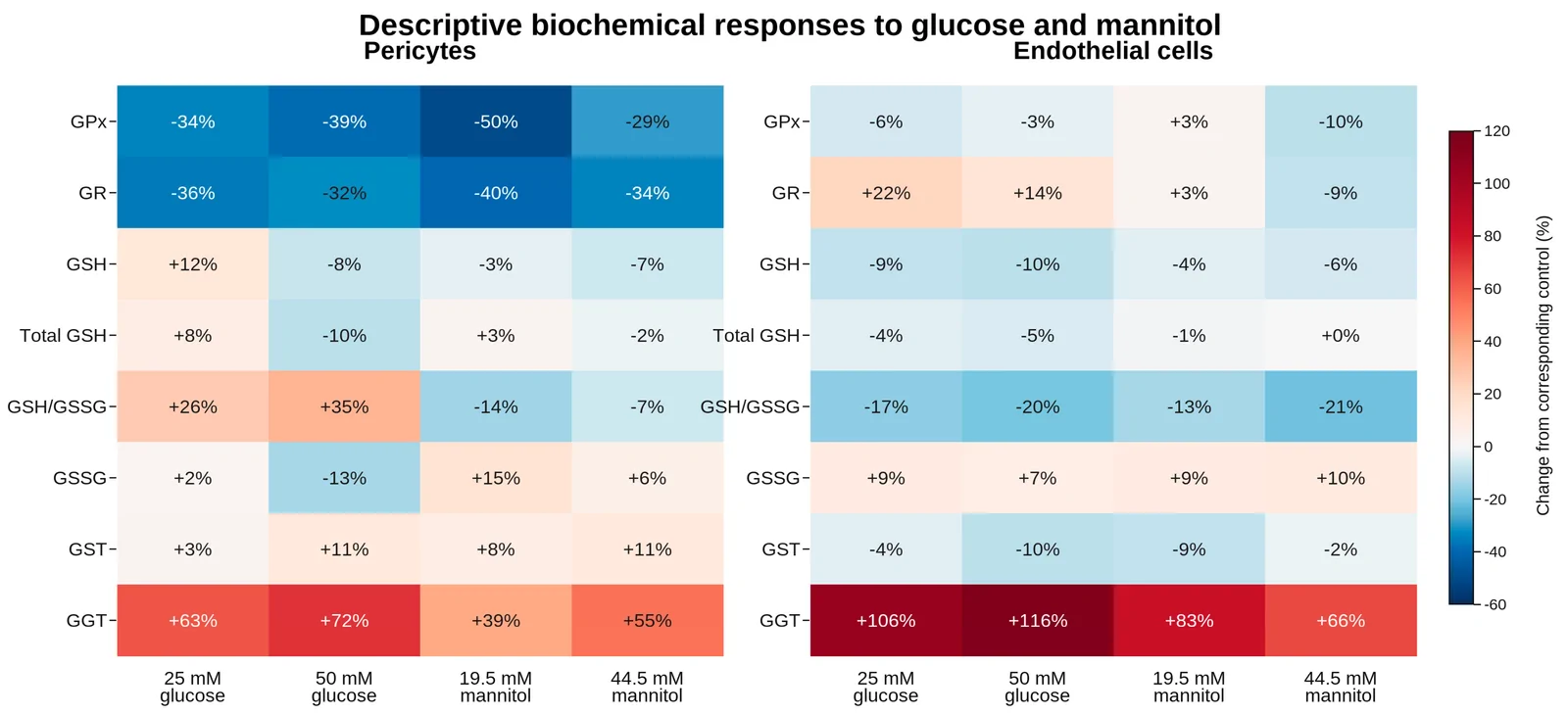
Percentage change from the corresponding control across experimental conditions. Note: Color encodes percentage change relative to the corresponding control mean within each experiment. Values are descriptive and are not inferential tests.

The principal pericyte findings were lower GPx and GR activity together with higher GGT (Table 1 and Figure 1). GPx showed a large condition-associated effect (eta squared = 0.393): relative to control, activity was 34% lower at 25 mmol/L glucose, 39% lower at 50 mmol/L glucose, and 50% lower with 19.5 mmol/L mannitol; the 29% reduction with 44.5 mmol/L mannitol was not statistically significant. Neither glucose condition differed from its nominally matched mannitol comparator, so the GPx pattern did not distinguish a glucose-specific response from mannitol-associated hyperosmotic stress.

**Table 1:** Biochemical endpoints across experimental conditions in pericytes (10 technical replicate wells per group; one culture experiment).

Marker	Control	G25	G50	M19.5	M44.5	Welch *p*	η^2^
**GPx**	881.81 ± 196.08	579.75 ± 214.09	539.15 ± 175.21	441.72 ± 101.79	623.60 ± 245.47	**<0.001**	0.393
**GR**	446.59 ± 67.37	284.03 ± 117.43	301.89 ± 100.86	269.74 ± 65.47	292.96 ± 55.35	**<0.001**	0.393
GSH	387.58 ± 31.74	433.20 ± 95.08	355.99 ± 35.67	374.98 ± 13.31	360.90 ± 24.38	0.071	0.260
Total GSH	602.82 ± 57.97	651.73 ± 183.73	543.77 ± 120.82	622.37 ± 86.80	588.27 ± 109.67	0.503	0.092
GSH/GSSG	1.96 ± 0.69	2.48 ± 1.07	2.64 ± 1.57	1.69 ± 0.57	1.82 ± 0.69	0.226	0.137
GSSG	215.24 ± 63.14	218.53 ± 128.62	187.78 ± 109.02	247.39 ± 85.64	227.37 ± 93.21	0.763	0.041
GST	74.71 ± 13.66	77.19 ± 13.12	82.98 ± 15.78	80.77 ± 12.47	82.70 ± 7.46	0.529	0.066
**GGT**	7.60 ± 1.54	12.40 ± 1.59	13.05 ± 3.01	10.54 ± 2.24	11.75 ± 1.33	**<0.001**	0.498

Note: Mean ± SD. Units: GPx, GR, and GST, nmol/s·L; GGT, U/L; glutathione concentrations, µmol/L; GSH/GSSG, unitless. Control, 5.5 mmol/L glucose; G25/G50, 25/50 mmol/L glucose; M19.5/M44.5, control plus 19.5/44.5 mmol/L mannitol. Shading marks the principal within-experiment findings. η^2^ = one-way eta-squared effect size, interpreted descriptively for technical wells; ω^2^ (omega squared) is not reported. Data in bold are statistically significant.

GR showed a similarly large condition-associated effect (eta squared = 0.393) and was 32–40% lower than control in all four treatment groups. Again, neither glucose condition differed from its matched mannitol comparator, indicating that the reduction was shared across metabolic and hyperosmotic exposures within this experiment.

GGT was the largest pericyte responder (eta squared = 0.498), increasing by 39–72% relative to control across all four treatment conditions. The matched glucose–mannitol contrasts were not significant, suggesting a stress-associated response common to both types of exposure. GST, total GSH, GSSG, and GSH/GSSG did not show significant group effects. Reduced GSH yielded discordant Welch and Kruskal–Wallis results and was therefore considered inconclusive rather than definitively altered.

### 3.2. Endothelial Response Profile

The dominant endothelial finding was the GGT response (Table 2 and Figure 1), which was also the strongest treatment-associated effect in the dataset (eta squared = 0.763). Relative to control, GGT increased by 106% at 25 mmol/L glucose, 116% at 50 mmol/L glucose, 83% with 19.5 mmol/L mannitol, and 66% with 44.5 mmol/L mannitol (all adjusted *p* < 0.001). Most importantly, GGT was 30% higher at 50 mmol/L glucose than in the nominally matched 44.5 mmol/L mannitol condition (adjusted *p* = 0.009). This was the clearest indication of a possible glucose-specific effect in the experiment.

**Table 2:** Biochemical endpoints across experimental conditions in endothelial cells (10 technical replicate wells per group; one culture experiment).

Marker	Control	G25	G50	M19.5	M44.5	Welch *p*	η^2^
GPx	1914.64 ± 245.98	1809.09 ± 421.55	1854.56 ± 274.83	1969.86 ± 483.20	1727.89 ± 370.42	0.696	0.054
GR	1045.02 ± 158.72	1271.88 ± 434.46	1186.14 ± 378.21	1080.74 ± 281.98	953.91 ± 270.42	0.346	0.118
GSH	406.56 ± 52.72	369.72 ± 27.30	365.73 ± 11.17	389.57 ± 14.28	381.14 ± 21.87	0.005	0.216
Total GSH	568.37 ± 56.41	546.23 ± 36.94	540.94 ± 24.36	564.01 ± 33.14	568.18 ± 47.73	0.306	0.081
GSH/GSSG	2.65 ± 0.73	2.19 ± 0.59	2.13 ± 0.34	2.31 ± 0.43	2.09 ± 0.35	0.296	0.147
GSSG	153.94 ± 25.50	167.39 ± 23.52	164.86 ± 25.26	167.28 ± 23.93	169.18 ± 18.11	0.670	0.057
GST	71.40 ± 10.59	68.37 ± 7.56	63.95 ± 9.08	65.06 ± 3.72	70.02 ± 9.91	0.259	0.110
**GGT**	7.65 ± 1.10	15.76 ± 2.08	16.50 ± 2.20	13.99 ± 1.24	12.68 ± 2.25	**<0.001**	0.763

Note: Mean ± SD. Units: GPx, GR, and GST, nmol/s·L; GGT, U/L; glutathione concentrations, µmol/L; GSH/GSSG, unitless. Control, 5.5 mmol/L glucose; G25/G50, 25/50 mmol/L glucose; M19.5/M44.5, control plus 19.5/44.5 mmol/L mannitol. Shading marks GGT, the principal within-experiment finding. η^2^ = one-way eta-squared effect size, interpreted descriptively for technical wells; ω^2^ (omega squared) is not reported. Data in bold are statistically significant.

Reduced GSH showed a smaller overall condition-associated effect (eta squared = 0.216), with mean reductions of 4–10% relative to control. However, neither high-glucose group differed significantly from its own control or matched mannitol comparator. The only pairwise difference involved the unmatched 50 mmol/L glucose and 19.5 mmol/L mannitol groups and was therefore treated as secondary exploratory evidence. GPx, GR, total GSH, GSH/GSSG, GSSG, and GST showed no significant group effects.

### 3.3. Candidate Endpoints for Confirmatory Studies

GGT showed marked treatment-associated increases in each of the two separate datasets. This recurrence does not establish a common biological effect or a difference between cell types because the experiments were unmatched. Instead, it identifies GGT as the principal candidate endpoint for independent replication under a matched experimental design.

Within the pericyte experiment, the GPx and GR reductions identify additional candidate endpoints, although neither enzyme distinguished high glucose from nominally matched mannitol exposure. Within the endothelial experiment, the 30% higher GGT at 50 mmol/L glucose than at 44.5 mmol/L mannitol identifies the highest-priority contrast for replication.

### 3.4. Supporting Exploratory Pairwise Comparisons

Table 3 preserves the exploratory Games–Howell comparisons for transparency. Among the nominally matched glucose–mannitol contrasts, only endothelial GGT at 50 mmol/L glucose differed from the 44.5 mmol/L mannitol condition. These calculations describe separation among technical wells within this experiment; they do not estimate between-experiment variability, establish reproducibility, or support population-level inference.

**Table 3:** Exploratory Games–Howell comparisons among technical replicate wells.

Cell Type	Marker	Comparison	Mean Difference	95% CI	Adjusted *p*
Pericytes	GPx	Control vs. G25	302.054	24.232 to 579.876	0.029
Pericytes	GPx	Control vs. G50	342.653	90.883 to 594.423	0.005
Pericytes	GPx	Control vs. M19.5	440.091	221.352 to 658.830	<0.001
Pericytes	GR	Control vs. G25	162.557	29.596 to 295.518	0.014
Pericytes	GR	Control vs. G50	144.694	26.921 to 262.467	0.013
Pericytes	GR	Control vs. M19.5	176.850	87.015 to 266.685	<0.001
Pericytes	GR	Control vs. M44.5	153.626	69.928 to 237.324	<0.001
Pericytes	GGT	Control vs. G25	−4.803	−6.918 to −2.688	<0.001
Pericytes	GGT	Control vs. G50	−5.457	−8.810 to −2.104	0.001
Pericytes	GGT	Control vs. M19.5	−2.938	−5.575 to −0.301	0.025
Pericytes	GGT	Control vs. M44.5	−4.150	−6.102 to −2.198	<0.001
Endothelial cells	GSH	G50 vs. M19.5	−23.833	−41.272 to −6.394	0.005
Endothelial cells	GGT	Control vs. G25	−8.112	−10.441 to −5.783	<0.001
Endothelial cells	GGT	Control vs. G50	−8.857	−11.300 to −6.414	<0.001
Endothelial cells	GGT	Control vs. M19.5	−6.340	−7.932 to −4.748	<0.001
Endothelial cells	GGT	Control vs. M44.5	−5.036	−7.527 to −2.545	<0.001
Endothelial cells	GGT	G25 vs. M44.5	3.076	0.144 to 6.008	0.037
Endothelial cells	GGT	G50 vs. M19.5	2.517	0.032 to 5.002	0.047
Endothelial cells	GGT	G50 vs. M44.5	3.821	0.813 to 6.829	0.009

Note: Mean difference = first group minus second group. Adjusted *p* values are from exploratory Games–Howell comparisons among technical replicate wells from one culture experiment. They quantify within-experiment separation among wells only and cannot estimate biological variability, demonstrate reproducibility, or support inference to a wider population.

## 4. Discussion

This exploratory study identified candidate responses within two separate culture experiments. In the pericyte experiment, GPx and GR were lower and GGT was higher across several treatment conditions. In the endothelial experiment, GGT was the dominant response, and its value at 50 mmol/L glucose exceeded that at nominally matched 44.5 mmol/L mannitol by 30%. These observations do not constitute evidence of differences between cell types because technical wells were derived from single, unmatched experimental runs.

### 4.1. Coordinated GPx and GR Reduction in Pericytes

GPx and GR form a coupled peroxide-detoxification and glutathione-recycling system: GPx consumes GSH while reducing peroxides, and GR uses NADPH to regenerate GSH from GSSG. The coordinated 32–50% reductions observed in pericytes may therefore reflect diminished catalytic reserve, altered enzyme regulation, or substrate and cofactor constraints during acute stress. Similar changes in antioxidant pathways have been reported in retinal pericytes exposed to diabetic conditions [8,16,17]. However, total GSH, GSSG, and GSH/GSSG remained statistically stable, so these data support enzyme-level remodeling rather than demonstrable depletion of the measured glutathione pool after 24 h. Because activity was measured in a combined medium-cell-lysate preparation and was not normalized to biomass, intracellular enzyme regulation cannot be inferred directly.

The parallel reductions under high glucose and nominally matched mannitol are equally informative: within this experiment, GPx and GR suppression was not glucose-specific. Hyperosmotic signaling, cell-volume regulation, or direct mannitol effects may account for part or all of the response [19,20]. Direct osmolality measurements, an additional osmotic agent, and iso-osmolar media formulations would be needed to separate these possibilities.

### 4.2. GGT-Mediated Glutathione Turnover and the Endothelial Glucose–Mannitol Contrast

GGT showed marked treatment-associated increases in each separate experiment. As an ectoenzyme, GGT initiates extracellular glutathione catabolism by cleaving the gamma-glutamyl bond of GSH and GSH conjugates, generating cysteinylglycine and gamma-glutamyl products. Subsequent processing releases precursor amino acids, particularly cysteine, that can be taken up and reused for intracellular GSH synthesis [11,12,13,14]. The increases may therefore indicate accelerated extracellular glutathione turnover or greater demand for precursor salvage during metabolic or hyperosmotic stress. This interpretation concerns a candidate pathway and not a demonstrated flux mechanism.

An increase in GGT activity should not, however, be interpreted as uniformly protective. Enhanced salvage could help sustain intracellular GSH, but GGT-dependent cysteinylglycine formation can also support pro-oxidant reactions in the presence of redox-active metals [13,14]. Stable total GSH and GSSG concentrations do not exclude altered flux through this pathway because steady-state pool sizes can be maintained despite faster turnover. Moreover, the combined medium-cell-lysate assay cannot distinguish membrane-associated GGT from enzyme released into the medium, nor can it determine whether the change reflects GGT1 expression, catalytic activation, cell abundance, or altered substrate availability.

The 30% higher endothelial GGT at 50 mmol/L glucose than at nominally matched 44.5 mmol/L mannitol is the clearest evidence for a possible glucose-specific component. Because the added solute concentrations were matched nominally, this contrast suggests that glucose metabolism or glucose-responsive signaling contributed beyond osmotic exposure alone. Hyperglycemia can increase mitochondrial and enzymatic reactive oxygen species production and engage glycation, protein kinase C, and inflammatory pathways that could increase glutathione use, export, or precursor demand [1,2,3,4,5,18]. The present experiment did not measure these pathways, however, and it did not confirm medium osmolality; therefore, endothelial GGT should be regarded as a candidate glucose-sensitive response rather than proof of a defined mechanism.

The smaller endothelial GSH effect does not provide parallel evidence for glucose specificity because neither high-glucose condition differed from its matched mannitol group. The main endothelial interpretation consequently rests on GGT, not on depletion of the measured glutathione pool.

### 4.3. Core Contribution and Limits of Inference

The core contribution of this study is the endpoint and contrast prioritization within a defined five-condition framework, not a comparison between cell types. The large GGT responses identify this enzyme as the main target for confirmatory work, and the endothelial 50 mmol/L glucose versus 44.5 mmol/L mannitol contrast identifies a specific test of a possible glucose-related component. The pericyte GPx and GR reductions provide secondary targets for replication and for determining whether their suppression reflects osmotic stress, glucose-related stress, or both.

The current data cannot separate cell identity from culture medium, experimental run, baseline activity, or unmeasured cell abundance. Absolute values and response shapes must therefore remain within-experiment descriptions. A valid comparison between cell types will require independent biological repeats performed in matched runs, biomass normalization, and a formal cell-type-by-condition interaction analysis.

### 4.4. Strengths, Limitations, and Future Directions

The main strength of this exploratory study is its structured glutathione-centered screening under a defined five-condition framework within each separate culture experiment. The eight-endpoint panel sampled antioxidant defense, glutathione recycling, conjugation, and extracellular salvage. Nominally concentration-matched mannitol conditions helped identify responses shared with hyperosmotic exposure and one contrast that may contain a glucose-related component. The analysis therefore provides a focused set of endpoints and contrasts for a prospectively replicated study.

The limitations remain substantial. Technical wells from a single experiment cannot substitute for independent biological replication, and the unmatched runs preclude comparison between cell types. No Calcein AM or other quantitative viability assay was performed, medium osmolality was not measured, and assay values were not normalized to protein, DNA, or viable cell number. The findings should consequently be treated as preliminary evidence for study planning rather than confirmation of a reproducible biological effect.

Future work should extend the monoculture, 24 h model to multiple exposure durations, physiologically graded glucose concentrations, and co-culture or microfluidic blood–retinal barrier systems that capture pericyte–endothelial crosstalk.

Direct measurement of medium osmolality and inclusion of an additional osmotic comparator would clarify which responses reflect hyperosmotic stress rather than mannitol-associated effects.

Parallel assessment of viability, cell number, and total protein would distinguish enzyme regulation from changes in cell abundance and would strengthen comparisons across experiments.

Adding cytotoxicity, reactive oxygen species, mitochondrial function, and inflammatory signaling endpoints would provide mechanistic context for the observed glutathione signatures.

Because total GSH and the GSH/GSSG ratio are mathematically related to the reduced and oxidized glutathione measurements, future multivariable analyses should account for this dependence rather than treating all eight endpoints as independent dimensions.

Independent biological repeats will be required to estimate between-experiment variability and to test cell-type-by-condition interactions directly.

Finally, targeted transcript and protein measurements of GCLC/GCLM, GSR, GP_X_ isoforms, GST isoforms, and GGT1 could determine whether the observed activity patterns arise from altered expression, enzyme inhibition, or substrate limitation.

## 5. Conclusions

This exploratory screen identifies GGT as the principal endpoint for independent biological replication. GGT increased across treatment conditions within each separate experiment, and endothelial GGT at 50 mmol/L glucose exceeded the nominally matched 44.5 mmol/L mannitol condition, defining a candidate glucose-sensitive contrast. The pericyte experiment additionally identified GPx and GR suppression as targets for follow-up. The study does not support comparisons between cell types. Matched independent experiments with biomass normalization, viability assessment, and direct osmolality measurement are required before reproducible or mechanistic conclusions can be drawn.

## Data Availability

The data presented in this study are available on request from the corresponding author due to an ongoing patent application process and the need to protect the associated intellectual property. The data may be made available following completion of the patenting process, subject to applicable intellectual property restrictions.

## Abbreviations

The following abbreviations are used in this manuscript:

DR	diabetic retinopathy
GPx	glutathione peroxidase
GR	glutathione reductase
GSH	reduced glutathione
GSSG	oxidized glutathione
GST	glutathione S-transferase
GGT	γ-glutamyl transferase
